# Cookery

**Published:** 1862-03

**Authors:** 


**COOKERY.**—There can be no doubt that health iss ometimes undermined, and life itself lost, by bad cookery. The following items, gathered from reliable exchanges, are well worthy of attention.

**BREAD WITHOUT YEAST OR DRUGS.**—Bread can be made light, wholesome, and palatable to the unperverted taste, without rotting by fermentation, or poisoning with saleratus, cream of tartar, etc., in the following manner: Take cold water, the colder the better—ice-water is the best—stir in *unsifted* wheat-meal, enough to make a batter not very stiff; stir quickly while adding the meal, so as to introduce all the air possible. Put it in small patty-pans (cake-tins)—these are better than large dishes—and bake in a *hot* oven, hotter than for any other bread. Bake it half an hour or more. A little experience in making and baking will convince any one that bread can be made *light* without yeast or "lightening" of any kind, except air and water; and those who regard *good* bread as the staff of life will ask no better. If any should not succeed the first time, *try again*, for it can be done. The baking is the nost important part of the operation; the oven must be *hot.*

The following directions for making bread were given by the ladies to whom premiums were awarded for the best samples shown at the Presque Isle (Me.) Agricultural Exhibition. Mrs. C. P. Bean says: "I take one and a half cupfuls of new milk, and the same amount of boiling water, and add flour to this to make yeast, and let it set till it rises; then add flour until the dough is thick enough for baking. Then let it rise one half hour; then bake it."

Mrs. Sarah A. Emerson's method: "Take one pint of boiling water, one half tea-spoonful of salt; when it is lukewarm, stir in flour until it becomes thick batter; set the dish in warm water, in a warm place, until the batter rises. Then mix with it one quart of sweet milk or water; stir in flour until it forms a thick batter; set it in a warm place until it rises; add flour until is hard enough to knead; then let it set until it rises again, and bake it by a gradual fire until done."

**BAKED BEANS.**—Few people know the luxury of baked beans, simply because few cooks properly prepare them. Beans, generally, are not cooked half long enough. This is our method: Two quarts of middling-sized white beans, two pounds salt pork, and one spoonful of molasses. Pick the beans over carefully, wash, and add a gallon of boiling hot soft water; let them soak in it over night. In the morning put them in fresh water, and boil them gently till the skin is very tender and about to break. Take them up dry, and put them in your dish; stir in your molasses, gash the pork, and put it down in the dish, so as to have the beans cover all but the upper surface; turn in boiling water till the top is just covered; bake with a steady fire four or five hours. Watch them, and add more water from time to time as it dries away. The molasses may be omitted.

**BUCKWHEAT-CAKES.**—Take about two quarts of water and one pint of milk, mixing in the buckwheat-meal, and about half a pint of brown flour, (the "middlings" of wheat.) This, we think, makes them much better than all buckwheat. Stir in two table-spoonfuls of salt, two large table-spoonfuls of good hop-yeast, beat well, and when of the desired thickness, cover and set the batter in a warm place, if in cold weather, to rise, and by breakfast-time, next morning, they will be up to the top of the kettle. We leave from a pint to a quart of the batter in the kettle after each baking, to raise the next one—it not being necessary to make them with fresh yeast more than two or three times during the winter. To this batter we pour the water, milk, and meal, as before, for the next batch. When we do not wish to have them for tea, we pour cold water over the batter remaining in the kettle, and set it away in a cool place, to keep it from becoming sour, and pour the water off when we wish to mix them again. Too much milk would have a tendency to sour them, and also makes them more difficult to bake; but used in moderate quantities, it is a great improvement to them, both in taste and appearance.

**RICE-PUDDING WITHOUT EGGS.**—Wash a half pound of rice, and put it in a broad, shallow tin-pan holding four quarts, ( we have a large family,) with a large tea-cupful of sugar, and a half tea-spoonful of salt. Fill the pan np with milk, fresh from the cow is best, and set in the oven or stove to bake, stirring it occasionally and trying the rice. When the latter is soft and begins to thicken the milk, the puddiug is done. If it boils too long, or there is too much rice in it, it will be too thick to be good.

**BEEFSTEAK**—Should be cut nearly an inch in thickness, and divided, by the natural divisions where practicable, into pieces the size of your hand, or thereabouts. Cut away the most of the fat.

The best gridiron is the double one of wire, which you can shut your meat into and turn without a fork to let the juice out; but any gridiron will do if it is clean. The outside of a broiled piece of meat must be crisp, and (*turn it*) the inside juicy, to make it the most palatable and (*turn it*) nourishing. If you allow it to rest long with one side to the fire, (*turn it*,) the juice and flavor rise to the surface and is lost. The great art (*turn it*) is to expose the meat at the start, for a moment, to such an intense heat, that (*turn it*) the several fibers may be seared in such a manner as to seal up—so to speak—the moisture. (*Turn it.*) Steak can be cooked in this way until it will not look bloody when cut, and (*turn it*) will satisfy fully those who like "rare" beef, without offending (*turn it*) such as prefer it "well done." Butter is worse than wasted—of course (*turn it*) you'll have it on the table for such as wish to disguise the taste of beef, as well as pepper and salt. (*Turn it.*) Your motto is, beef and fire. If your fire is a hot one, the steak is nearly done. Give the steak your entire attention, and turn it constantly.

**BEEF-STEW.**—A very economical and most savory and delicious dish can be made with two or three pounds of chuck-steak, (a cheap part of beef,) which infinitely surpasses the tasteless, insipid, common eating-house stuff called "beef *à la mode*." Cut the steak into pieces about two inches square; put them into a sauce-pan, with a large breakfast-cup of cold water; put it on the fire; as soon as it boils up, stand it on the hole to simmer for two hours until perfectly tender. While simmering, tie up, with a bit of thread or cotton, a bunch of herbs, composed of knotted marjoram, winter savory, and a little thyme; take it out just before the dish is served. Of course the stew must be occasionally shaken, as all others are; remember, however, the fat must not be skimmed off; the more fat there is the better is the stew. This dish is of Italian origin, and in that country is eaten with plain, boiled maccaroni and Parmesan cheese, or with salad; and with either it is a "dainty dish to set before a king." Any girl from a charity-school could cook it, while an alderman of Portsoken ward, and a three-stone man, or a cripple from the work-house, would equally enjoy it, and wish he could eat more.

**HOMINY.**—After the hominy is well washed, instead of putting it into an open pot or kettle to boil, as is the usual practice, get a tin kettle of the size wanted, put the same into a common iron pot that will hold about one third more, which will leave a space around the tin to be filled with water. Then put the hominy into the tin kettle, with a suitable quantity of water, fill the pot pretty full of water, put the lids on the kettle and the pot, and let the hominy boil upon the stove, stirring it two or three times while boiling. By so doing, it will be found that the quality of the article will be much improved; more than half the usual work of stirring and tending will be saved, together with a large part of the work in cleaning the kettle after using, which has heretofore been the chief objection to cooking this dish. The tin kettle should be kept from touching the bottom of the pot, by means of a large wire, crooked for the purpose, and laid in the bottom, so as not to have the tin and iron come in contact while boiling. By this means, none burns to the kettle, and the burnt flavor, which is so noticeable in that cooked in the old-fashioned way, is entirely avoided.

**WATER-PROOF GLUE.**—Fine shreds of India-rubber, dissolved in warm copal varnish, make a water-proof cement for wood and leather. Take glue, twelve ounces, and water sufficient to dissolve it; then add three ounces of resin, and melt them together, after which add four parts of turpentine. This should be done in a water-bath or in a carpenter's glue-pot. This also makes a very good water-proof glue.

**"SPAULDING'S GLUE SUPERSEDED."**—The *American Agriculturist* recommends the following preparation for mending almost all articles that can be "stuck" together. It is named "Diamond Cement," and is often sold under that name at twenty-five cents for a two-ounce vial: Take one pound white glue; one quarter pound white lead, (dry;) one quart rain-water; one half pint alcohol. Place the first three ingredients in a kettle, and set the kettle in a dish of water. Boil it until the glue is dissolved; then add the alcohol, and boil again until all is well mixed. Keep it in well-stopped bottles. Use it in the same manner as glue. Should it be a little hardened when wanted for use, soften it by placing the bottle in warm water.

**MUSH BISCUIT.**—Make about a quart of Indian-meal mush or stirabout; while hot, add a piece of butter, about the size of an egg, thin it with milk, adding a little salt; then add some flour, thin it with a tea-cup of yeast, then add as much more flour as will make it the consistence of dough; knead it well, set it to rise, and bake with a hot fire. The meal makes the bread light, and thus removes the objection to the unhealthfulness of hot bread.

